# Between Care and Mental Health: Experiences of Managers and Workers on Leadership, Organizational Dimensions, and Gender Inequalities in Hospital Work

**DOI:** 10.3390/healthcare13101144

**Published:** 2025-05-14

**Authors:** Elisa Ansoleaga, Magdalena Ahumada, Elena Soto-Contreras, Javier Vera

**Affiliations:** 1 Faculty of Psychology, Universidad Diego Portales, Santiago 8370067, Chile; 2 Faculty of Psychology, Alberto Hurtado University, Santiago 8340576, Chile; mahumadam@uahurtado.cl; 3 Department of Psychology, University of Concepción, Concepción 4070409, Chile; elenabsoto2016@udec.cl; 4 Independent Researcher, Santiago 7800251, Chile; javerab@gmail.com

**Keywords:** leadership, occupational health, psychosocial risks, gender inequality, recognition, role stress, hospital work, mental health, healthcare workers

## Abstract

Work is a key social determinant of mental health, and adverse organizational conditions in healthcare settings increase psychosocial risks. Leadership influences workplace well-being, yet its impact on mental health and gender inequalities remains underexplored. Despite the feminization of the health sector, disparities persist in leadership access, role expectations, and work–family reconciliation, exacerbating occupational stress. **Aims:** This study examines leadership practices in public hospitals, focusing on their relationship with mental health, organizational dimensions (recognition and role stress), and gender disparities. It explores the perspectives of both workers and managers to understand how leadership shapes workplace conditions and well-being. **Methods:** A qualitative, cross-sectional study was conducted as part of the FONDECYT project 1220547. Semi-structured interviews were conducted with 64 workers from public hospitals in Santiago, Chile, including clinical and administrative staff. The analysis supported by Grounded Theory identified key categories: constructive and destructive leadership, recognition, role stress, and gender disparities in leadership. **Results:** Constructive leadership—characterized by communication, fairness, and recognition—was linked to a healthier work environment and improved well-being. In contrast, destructive leadership (characterized by abuse of power and imposition, or inaction, lack of support, and absence of effective direction) contributed to role stress, workplace mistreatment, and job dissatisfaction. Recognition was a crucial but insufficient motivator, as the lack of formal mechanisms led to frustration. Role stress emerged as a significant risk for well-being, with subordinates experiencing overload, ambiguity, and conflicting expectations. Gender inequalities persisted as women faced more tremendous barriers to leadership and difficulties balancing work and family responsibilities. Workers and managers had differing perspectives, with subordinates prioritizing fairness and recognition while managers emphasized operational constraints. **Conclusions:** Leadership training should emphasize trust, equity, and recognition to enhance workplace well-being. Institutional policies must address role stress, strengthen formal recognition systems, and promote gender equity in leadership. Future research should integrate quantitative methods to explore leadership’s impact on organizational conditions and mental health outcomes.

## 1. Introduction

Work is a key social determinant of mental health. Evidence indicates that both adverse working conditions and organizational dimensions influence depressive symptoms and psychological distress. Factors such as destructive leadership [1,2], workplace violence [3], and job vulnerability [4,5] have been widely documented. In addition, organizations reproduce social inequalities [6], which generate differentiated impacts on workers’ mental health.

One of the most studied inequalities in the labor sphere is gender inequality. The sexual division of labor increases women’s vulnerability in this context [7], exposing them to higher levels of precariousness [8,9] and more frequent mental health problems such as distress and psychotropic use [10,11,12]. However, women’s occupational risk conditions have been less studied compared to those of men [13].

This scenario is particularly evident in the health sector, where emotional and organizational demands increase psychosocial risk [1,14,15,16,17]. In Chile, 57% of occupational diseases correspond to mental health pathologies [18], with the health sector being one of the three with the highest prevalence. The COVID-19 pandemic made these risks even more visible [19].

Among the organizational dimensions that affect mental health are recognition and role clarity. Recognition is usually analyzed based on the balance between the effort or demands imposed on the worker and the rewards they receive, such as salary, social recognition, and professional development. Studies of health workers indicate that when workers deploy great efforts and, in return, receive few rewards, they report deterioration of mental well-being, burnout, and musculoskeletal disorders, among others [20,21,22,23]. On the other hand, problems derived from role clarity include role overload (when demands exceed available time), role ambiguity (confusing or inconsistent expectations), and role conflict (incompatible expectations). These subdimensions have been linked to emotional exhaustion, depersonalization, and impaired psychological well-being [24,25].

Although psychosocial risks in the health sector have been widely studied, their impact is not gender neutral. Even though the health industry is highly feminized, women hold around 70% of healthcare worker jobs worldwide [26], and women occupy lower-status and lower-remuneration positions. At the same time, men predominate in strategic and leadership positions [27,28]. In addition, female health workers report a higher prevalence of mental health problems associated with emotional demands and workplace violence [29,30].

Leadership is a central organizational factor that can affect workers’ mental health, whether through destructive practices [2,31,32], tolerance, perpetuation of violence [33], or omission in the design and organization of work [34,35]. In addition, the interaction between leadership and social dimensions, such as gender and power, has been little explored [36,37]. An example is the persistent gender gap in leadership positions within the health sector [38], without significant efforts to address these inequalities [39]. In Latin American and Caribbean countries, women hold less than 30% of leadership positions in healthcare. A Women in Global Health report also illustrates that women occupy only 25% of senior leadership roles [26,40], even though the positive impact of women’s leadership in the health sector and global health has been documented [41].

Einarsen et al. [33] have developed a conceptual model of constructive–destructive leadership that is widely used in the study of leadership. This model defines destructive leadership as a systematic pattern of behavior that undermines organizational goals and workers’ well-being. However, the same leader can simultaneously exhibit constructive and destructive behaviors [42].

Promoting inclusive and well-being-oriented leadership is fundamental to improving the work environment in healthcare. However, studies on leadership in this sector have focused on its relationship with service quality, efficiency, and innovation [43,44,45], leaving aside its impact on workers’ health. A key aspect in promoting effective leadership is trust, which is understood as interdependence based on the perception of benevolence, honesty, openness, reliability, and competence [46]. These qualities strengthen the organizational climate and are essential in leadership management.

Considering the psychosocial risks of health work, the relevance of leadership, recognition, and role clarity as organizational factors, as well as the reproduction of social inequalities in organizations [6], this article aims to describe and analyze the practices and processes associated with healthcare leadership that may affect the mental health and well-being of workers, with emphasis on the organizational and social inequality dimensions in typical work contexts from the experience of workers and leaders.

## 2. Materials and Methods

### 2.1. Design and Approach

This study is part of the project Liderazgos Destructivos y Salud Mental en el Trabajo en Salud en Chile, financed by the Fondo Nacional de Desarrollo Científico y Tecnológico (FONDECYT, ANID, N°1220547). A mixed design (quantitative and qualitative) was adopted, and this article presents the findings of the qualitative phase.

The qualitative phase was based on a qualitative descriptive case study using a grounded theory approach. This strategy was used to analyze the practices and processes associated with health leadership in typical work scenarios, considering organizational dimensions and social inequality [47,48].

### 2.2. Participants and Sampling

The sample consisted of 64 workers from public hospitals in Santiago de Chile, including administrative and clinical staff, with representation from regular care areas and critical units. The selection criteria were sex (men and women) and hierarchical position (managers and subordinates) (see Table 1). More women were incorporated, aligning with the health sector’s feminization and the need to explore gender inequalities.

The sampling was purposive and snowball sampling, and participants with leadership experience or exposure to relevant organizational dynamics were included (See Table 2 and Table 3). The participants were invited to participate in this study, and those interested contacted the research team.

### 2.3. Data Collection Procedure

Semi-structured interviews were conducted between January and December 2024 in face-to-face and virtual formats. The interviews were recorded with informed consent and had an average duration of 50 min. The sections of the interview guide are listed in Table 4.

### 2.4. Ethical Considerations

This study complied with the principles of the Declaration of Helsinki, guaranteeing anonymity, voluntariness, confidentiality, and informed consent. It was approved by the Ethics Committee of the Universidad Diego Portales (014-2022).

### 2.5. Data Analysis

The data were analyzed following the recursive principles of Grounded Theory, i.e., data collection and analysis are interrelated processes, and both descriptive analysis (open coding) and relational analysis (axial and selective coding) were performed [49]. An iterative process was carried out to analyze new data and clarify information to achieve theoretical saturation. Furthermore, the density of each category was checked to identify its properties. Finally, the categories were checked to ensure they were mutually exclusive.

Two researchers analyzed the results to reach a consensus on the categories. A third member of the research team resolved any disagreements.

The research team transcribed the quotations, considering translation and back-translation processes to ensure greater data accuracy.

The analysis was performed with Atlas. Ti 24 software, ensuring methodological rigor and traceability of the findings.

This structure enabled the capture of narrative patterns and their interactions, facilitating a deep understanding of the study phenomenon.

## 3. Results

A combination of organizational factors, leadership styles, and gender dynamics determines healthcare workers’ well-being and mental health. The interaction between these dimensions shape both experiences of support and recognition and situations of stress, emotional exhaustion, and vulnerability.

From the analysis of the narratives, three central axes are identified in the relationship between leadership and workers’ mental health: the role of leadership in the perception of psychological support and security; the impact of role stress and recognition on mental health; and gender inequalities in access to and exercise of leadership and their effect on occupational well-being.

### 3.1. The Role of Leadership

#### 3.1.1. Constructive Leadership: Expressions and Practices

From the perspective of subordinates, constructive leadership translates into managers who establish effective two-way communication, encourage respectful treatment, and promote team development. Among the most valued behaviors, the following are identified:

“A good leader first has to communicate with his workers… Know how to listen to the workers…”

[Interview3—Administrative worker, man, subordinate].

Managers agree that positive leadership is based on effective communication, private feedback, and recognition of a well-done job. However, they also emphasize other aspects of organizational management, such as trust in workers and establishing conditions that allow their professional development. Managers’ narratives are reflected in these accounts:

“Good leadership, for example, would be a boss who supports you, who responds, who is with you, who gives you the tools, who listens to you, and who can respond or who can support you, give you options or alternatives, or teach you in case you need something”

[Interview12—Nurse, woman, manager].

Both subordinates and managers agree that positive leadership is based on trust, recognition, and constructive feedback. However, managers emphasize technical knowledge as a fundamental element of their role, while subordinates value closeness and organizational justice more.

**Figure 1:**
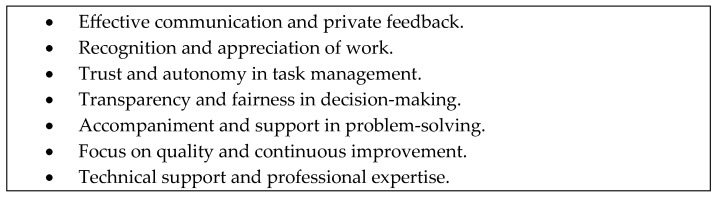
Summary of positive leadership characteristics.

The dimensions listed in Figure 1 reflect the expectations and experiences of the actors involved in hospital management, highlighting the relevance of leadership that balances operational management with worker well-being.

#### 3.1.2. Destructive Leadership: Expressions and Practices

From the perspective of subordinates, negative leadership in public hospitals manifests itself through behaviors that affect well-being and organizational dynamics. Workers’ testimonies allow us to identify two main types of negative leadership: authoritarian and laissez-faire. While the former is characterized by abuse of power and imposition, the latter is associated with inaction, lack of support, and absence of effective direction.

These behaviors generate work environments marked by tension, injustice, and frustration in the teams. Some narratives illustrate these experiences:

“…distant, directing from his office and only delivering orders without getting actively involved, but waiting for the occurrence of errors to punish workers”

[Interview3—Administrative worker, man, subordinate].

“There were no solutions… and the leadership was hiding… when they were mistreating us, and they were hiding in their office, locked up”

[Interview25—Administrative worker, woman, subordinate].

From managers’ perspectives, negative leadership is characterized by behaviors that affect team management and organizational climate. Two main dimensions are identified: authoritarian and inflexible leadership and evasive and ineffective leadership. While the first type of leadership is associated with the abuse of power and a lack of consideration for the team’s opinions, the second is linked to the omission of responsibilities and inaction in the face of conflicts. Some testimonies illustrate these experiences:

“…and she starts yelling at me and says, ‘Why do you have to give a reason to that manager who comes to get into our unit and give her opinion about the patients and she has no reason to be getting involved in the treatment, in the diagnosis?’”

[Interview36—Nurse, woman, manager].

“When it comes to resolving some conflicts or conversations that are somewhat uncomfortable, he avoids them. When he has to give feedback on something that may be uncomfortable, for some mistake that some physician on his team has made, he does not have those difficult conversations”

[Interview49—Kinesiologist, man, manager].

#### 3.1.3. The Role of Leadership in Perceived Psychological Support and Safety

Leadership is a key factor in the perception of psychological safety in work teams.

According to interviews with managers and subordinates, leaders’ experiences and practices can be categorized as constructive or destructive and can be associated with the harmful or salutogenic effect they can have on workers.

Positive leadership based on support, empathy, and recognition of the emotional burdens of working in health generates trust and an environment of well-being. Workers value leaders who are present in critical situations, validate work’s emotional burden, and foster a respectful and collaborative work environment.

On the contrary, when leadership is exercised in an authoritarian, distant, or negligent manner, it generates a perception of a lack of protection that increases work stress and emotional vulnerability. The absence of intervention in conflict situations and the normalization of abusive practices reinforce this feeling of insecurity and burnout.

“Knowing that my leader, in this case, the nurse, the shift manager, is there in the good times and in the hard times; and I am going to be able to turn to her”

[Interview17—Nurse Technician, man, subordinate].

“Look, for me, leadership has to be a person who is an example, who is a motivator as well, and who has emotional self-management.”

[Interview29—Physician, woman, manager].

### 3.2. Role Stress and Recognition

Leadership in public hospitals depends not only on the individual characteristics of managers but is also influenced by organizational dimensions that structure the work and condition the work experience of the teams. In this section, two key dimensions in the work organization of hospitals are analyzed: recognition at work and role stress, addressing both their positive and negative manifestations.

#### 3.2.1. Role Stress

Some employees value a clear structure in the delimitation of functions, as this facilitates collaboration with management and the willingness to take on additional tasks at critical moments. They also consider that flexibility in reassigning tasks and shifts is positive as it is communicated transparently and allows the team to participate:

“If I am already missing an extra shift or I cannot come to do it, one says to the colleague: ‘Could you do this extra shift that I have assigned?’ Then one tells the boss: ‘Boss, I cannot come to do an extra shift; I am going to get this person to do it.’ ‘Yeah, that is fine.’ Then she deletes it from the system and has the power to delete it from the system” [Interview9—Nurse Technician, woman, subordinate].

However, other workers report that function assignments and task changes occur without prior planning or adequate coordination, which generates uncertainty and affects work performance. In some cases, reassigning responsibilities responds more to emerging needs than an organized work design, making it challenging to manage workloads and generating confusion within teams:

“Now, what’s wrong with me? When the doctors, nurses, kinesiologists, and psychologists see me, they say, ‘Oh [Name omitted], I have a case for you’, and they bombard me with information or write me little notes and leave them in the office. The same goes for patients who feel they haven’t been referred to the doctor; they come knocking on the door saying, ‘Hello, I need to talk to you. Please, please, please, I need to talk to you. So there’s a structure for everyone else, but it doesn’t work for me”.

[Interview 57—Social Worker, Woman, subordinate].

The managers identify role stress as a key challenge in hospital management. Managers recognize the importance of establishing a clear work structure that defines and communicates the functions assigned to each role. Delegating administrative tasks is seen as a key strategy to enhance the clinical team’s efficiency, allowing them to focus on their primary duties without distractions.

“It is important that the shift manager is implemented so that colleagues are not taken out of their functions to do other administrative functions”

[Interview44—Nurse, woman, manager].

#### 3.2.2. Recognition

Subordinates highly value labor recognition since it directly influences the team’s motivation and performance. However, workers identify a series of deficiencies in the recognition mechanisms within the institution, highlighting their scarce formalization and the lack of organizational strategies to reinforce their application. The analysis of the narratives allows us to distinguish two key dimensions of job recognition:

- Formality of recognition: a distinction is made between formal recognition (institutionalized actions such as merit notes) and informal recognition (spontaneous thanks from bosses, colleagues, or patients);
- Source of recognition: various sources of recognition are identified, including managers, peers, and health system users.

“Yes, there are merit marks that come for care management, generally, but that is more than what your boss has, such that goes and makes you. No, it is more for the patients themselves filling out forms sometimes”

[Interview30—Nurse, woman, subordinate].

Managers consider labor recognition fundamental to strengthening trust, motivation, and commitment within the work teams. However, they emphasize that its implementation within public hospitals is deficient, especially in their work. In this regard, they identify three main dimensions of recognition:

- Recognition of workers: the importance of highlighting individual effort and contributions is appreciated, but it is recognized that this occurs in an informal and unsystematic manner;
- Lack of recognition for management: there is a perception of a lack of institutional protection since management receives little recognition from the organization;
- The imbalance between effort and reward in leadership roles: it is mentioned that there are no economic incentives or job stability for those who assume leadership responsibilities.

“At the moment, no other recognition. I mean, you know, here in the public system, we are managed by a single salary scale, basically, our grade, and I still have exactly the same grade as when I entered, and I was a clinical kinesiologist… So, obviously, being in charge of 30 people is not easy at all. Because of the level of decision-making and everything else, there is also merit at an economic level, but that has not materialized for the moment”

[Interview49—Kinesiologist, man, manager].

#### 3.2.3. The Impact of Role Stress and Recognition on Mental Health

Role stress and recognition are key organizational dimensions that influence the quality of work and the well-being of workers. Organizational conditions also impact work well-being, mainly through role stress and recognition.

- Role stress: work overload, ambiguity in the distribution of functions, and conflict between incompatible demands generate high stress levels and emotional exhaustion. Both subordinates and managers report that the lack of clarity in the assignment of roles and excessive workload contribute to fatigue and deterioration of mental health.

“[Overload is explained by]… someone did not come, someone was absent, so what does the shift manager have to do in this case? Restructure with what he has, and that is when, suddenly, certain problems may arise because some areas will be more lacking than others. So, the work that I do with three, I will have to do with two, or if there are two, I will have to do it alone, and that can put a little more pressure on me”

[Interview33—Nurse, woman, manager];

- Recognition: workers’ motivation and commitment are strengthened when their efforts are valued. However, the absence of formal recognition by the organization generates frustration and a sense of inequity, affecting job satisfaction and team morale.

“Mostly, we receive recognition from our users. The meaning of being given candy or chocolate or saying thank you is like gratification, but institutionally, no”

[Interview31—Nurse Technician, man, subordinate].

### 3.3. Leadership and Gender as a Dimension of Social Inequality

Subordinates’ narratives highlight three key dimensions of gender inequalities in hospital work:

- The perception of gender in the work environment and conflicts in mostly female teams: while some female workers recognize progress in protection against harassment and discrimination, some men perceive that the increasing regulation around gender equity has generated more significant conflict and competition in teams;
- Impact of maternity on employment decisions: maternity influences women’s career paths, determining their choice of positions and preference for certain institutions. Adjustments in work shifts are also needed. However, women workers perceive that organizational support in these circumstances is limited;
- Difficulties reconciling work and family life: working mothers report that exercising their rights, such as maternity leave or shift changes, can generate conflicts with their teams and work organization, contributing to a feeling of vulnerability and a lack of institutional support.

From the manager’s perspective, the health sector is widely recognized as a feminized space. However, there are differences in how this phenomenon is valued and its implications for management and leadership are perceived. The narratives of female managers highlight three key dimensions of gender inequalities in hospital work:

- Feminization of the sector and perceptions of the work environment: while some managers, especially women, consider that the high female presence contributes to a better understanding of the health needs of the population served, some men associate the predominance of women with a work environment marked by conflict and rumors;
- Difficulties of work–family reconciliation in leadership positions: female managers with children face problems like those of their female subordinates in reconciling work and family. However, they report that these situations can generate tensions within the team, especially when they involve the redistribution of shifts or adjustments in the workload;
- Persistence of gender stereotypes in access to leadership: despite the feminization of the sector, gender stereotypes continue to influence the perception of leadership, resulting in a preference for men in strategic or senior management roles.

#### Gender Inequalities in Access to and Exercise of Leadership and Their Effect on Well-Being

Although the health sector is a highly feminized space, the accounts show that there are still structural and cultural barriers that affect women’s professional careers and their well-being.

- Double workload and work-family reconciliation: women in managerial and operational positions face difficulties reconciling their work responsibilities with family demands. The need to change shifts or access maternity benefits can generate tensions in teams and a perception of disadvantage among those who must redistribute tasks;
- Gender perceptions of leadership: despite the high presence of women in the sector, leadership is still associated with traditionally male characteristics. This reinforces a structure where men occupy strategic positions while women are relegated to less recognized roles;
- Impact on mental health: the perception that women must constantly prove their competence in an environment where leadership remains masculinized generates additional pressure, increasing stress and burnout.

“There is also a social role in between gender stereotypes, and that influences. In other words, we have a culture in which we still think of men when we talk about leadership”

[Interview54—Administrative Worker, man, manager].

“Well, the only thing that really occurs to me is that since I am on maternity leave, which is basically the protection of the child up to the age of two, I have one hour of breastfeeding. So, uh, it was conflictive. In a low, low key, conflictive way, the fact that I must leave earlier and arrive later because that means that the person on duty has to stay longer”

[Interview56—Nurse, woman, subordinate].

## 4. Discussion

### 4.1. Synthesis of the Findings and Their Relation to This Study’s Objective

This study examined leadership practices and processes in public hospitals, considering their effects on mental health and their interaction with organizational factors and social inequalities. The results confirm that, in the vision of managers and subordinates, positive leadership based on recognition, effective communication, and fairness in task assignment contributes to a stable organizational climate and well-being at work. However, there are also differentiating aspects, since while managers highlight the importance of technical knowledge for the proper exercise of leadership, subordinates value the closeness of managers and the practice of organizational justice as key elements of positive leadership. In contrast, for managers and subordinates, negative leadership characterized by rigidity, unequal treatment, and avoidance of conflict confrontation increases insecurity and discomfort in teams, which aligns with previous research linking leadership and psychosocial risk [2,31,32].

Role stress and lack of recognition affect the perception of leadership and workers’ mental health. The effort–reward imbalance model warns that the absence of recognition in contexts of high work demand increases demotivation and burnout [20,21,50]. Ambiguity in the assignment of roles and work overload also emerge as critical factors that aggravate the negative perception of leadership and increase the risk of harassment and mistreatment [24,25]. There are differences in the narratives of subordinates and managers. Subordinates emphasize the need for recognition and equity in management, while managers highlight structural constraints and justify stricter practices for operational reasons. The effort–reward imbalance theory has been used to demonstrate the effects of the absence of recognition on workers’ health [51]. This study helps make the leader’s behavior [33] visible in mobilizing recognition (positively or negatively) at different levels (individual, group, and organizational), thus promoting or harming mental health.

Finally, the findings confirm the persistence of gender inequalities in the assignment of roles and performance evaluation in a highly feminized sector. In the literature linked to intersectionality, there are examples of how in health systems, the under-representation of women in leadership positions, as well as problems like the pay gap and physical and sexual violence, are all rooted in gender bias [52]. This study illustrates that women continue to face barriers to leadership access, greater performance demands, and difficulties reconciling work and family, and this last aspect is reflected both in the case of women in leadership roles and for subordinates. This reinforces the need for policies that promote gender equity and recognize the impact of stereotypes in the distribution of power in the hospital setting, which has even affected confidence in women’s leadership, solely based on biases [53]. Furthermore, research about leaders and leadership might collaborate to explore organizational mechanisms leading to intersectional invisibility and career inequity and co-create alternative mechanisms with policymakers [54].

### 4.2. Study Contributions

This study contributes to occupational health and hospital management by analyzing how leadership practices interact with organizational factors and social inequalities in public health. It integrates dimensions analyzed separately in previous studies, providing a broader view of their impact on occupational well-being.

- A comprehensive approach to health leadership: in contrast to previous research focused on service quality and organizational efficiency, this study examines the impact of leadership on workers’ mental health and well-being. It confirms that leadership can act as a protective factor or as a psychosocial risk, depending on its practices. Leadership that promotes trust and fairness generates healthier work environments, while negative leadership styles increase emotional burnout. Thus, leaders play a central role in fostering psychosocial safety, which, in turn, has been identified as a key dimension in fostering a high-functioning healthcare team culture [55];
- Interconnection between leadership, recognition, and role stress: leadership does not operate in isolation but is linked to organizational conditions that affect the work experience of workers. Firstly, the lack of formal recognition and incentives generates demotivation and emotional exhaustion, which aligns with the effort–reward imbalance model and mental health problems [51]. Secondly, there are critical dimensions of role clarity that negatively impact workers’ mental health, reinforcing the need to improve clarity in assigning roles within public hospitals;
- Gender perspective in hospital leadership: gender inequalities in a highly feminized sector are made visible. The findings show that women face more significant barriers to accessing leadership positions; female leadership is evaluated with more demanding criteria; and work–family reconciliation represents a considerable challenge for women, affecting their well-being and generating tensions in teams. This reinforces the urgency of implementing organizational policies that promote gender equity in leadership and facilitate work–family reconciliation. This is especially important in light of previous evidence showing the advantages for healthcare organizations of having women in leadership positions: ‘women leaders’ positive influence on six areas of impact: (1) financial performance, risk, and stability, (2) innovation, (3) engagement with ethical initiatives, (4) health, (5) organizational culture and climate outcomes, and (6) influence on other women’s careers and aspirations [41];
- Implications for hospital management and public policy: the findings directly impact hospital leadership management and occupational health policy design. Organizational leadership training is required to improve the management of well-being and equity in work teams. According to the effort–reward imbalance theory [51], formal recognition mechanisms must be implemented to prevent the perception of unrewarded effort. The distribution of roles and workloads should be reviewed to mitigate role stress and improve operational efficiency in hospitals.

### 4.3. Limitations of the Study

This study has some limitations that should be considered when interpreting the findings. First, although the qualitative approach allowed us to delve deeper into the workers’ experiences and perceptions, the results cannot be generalized to the entire population of healthcare workers in Chile. Second, since this study was conducted in public hospitals, the findings may not be fully extrapolated to other care settings, such as private health or primary care.

Another limitation is this study’s retrospective nature. The testimonies are based on workers’ experiences, which may imply memory or interpretation biases. Future research could complement these findings with quantitative methodologies to more fully assess the relationship between leadership, recognition, and role stress on healthcare workers’ mental health.

### 4.4. Implications and Projections for Prevention

The findings of this study have important implications for hospital management and the formulation of strategies to improve the well-being of healthcare workers. First, organizational efforts must promote formal recognition actions by leaders and develop strategies to minimize role problems. To this end, precise diagnoses on the distribution of roles and workloads are recommended, as is the adoption of sustainable interventions over time.

Strengthening leadership training in the health sector is also necessary, ensuring leaders develop skills in effective communication, conflict management, and promotion of team well-being. In this sense, healthcare institutions must move towards more inclusive and equitable leadership models that recognize and value the contributions of all workers, regardless of their gender or hierarchy.

Finally, this study highlights the need to design organizational policies that reduce gender gaps in access to leadership positions, making visible other dimensions that can complicate this relationship from an intersectional perspective, such as race, socioeconomic status, and age. Promoting diverse and well-being-oriented leadership is fundamental to ensuring a more equitable and healthy work environment in the health sector.

## 5. Conclusions

This article broadens the focus on leadership in the health sector, integrating organizational and social factors that have historically been studied in a fragmented manner. Its findings reinforce the idea that leadership cannot be analyzed only from the perspective of individual leaders but must be understood within an organizational and structural framework and social inequalities. The absence of recognition, ambiguity in the distribution of roles, and gender inequalities amplify the adverse effects of destructive leadership and cause harmful effects on the health and well-being of workers. Organizational management that promotes leadership based on trust, fairness, and team well-being is required, along with institutional policies that reduce work overload and foster recognition of health workers.

## Figures and Tables

**Table 1:** Sample characteristics.

Sample Characteristics			
Hierarchical Position	Man	Women	Total
Managers	16	16	32
Subordinates	10	22	32
Total	26	38	64

**Table 2:** Characterization of the participants according to sociodemographic and labor variables.

**Variable**	**n**	**%**
Sex		
Women	39	60.94%
Men	25	39.06%
Occupational hierarchy		
Managers	32	50%
Subordinates	32	50%
Setting		
Clinical	39	60.94%
Administrative	25	39.06%
Professional Category		
Medical	10	15.62%
Nursing	26	40.63%
Other healthcare professionals	11	17.19%
Administrative	8	12.50%
Technician	9	14.06%
Work Settings		
Intrahospital	40	62.50%
Outpatient	24	37.50%
Work Unit		
Critical Pacient Unit	34	53.12%
Regular Care	30	46.88%
Work Schedule		
Daytime	33	51.56%
Shift work	24	37.50%
Half day	3	4.69%
Missing	4	6.25%

**Table 3:** Characterization of the participants according to age and time in the job position.

Variables	M	SD	Minimum	Maximum
Age	37.91	8.95	26.00	62.00
Time in the job position (in months)	89.76	64.32	3.00	252.00

**Table 4:** Categories and subcategories for the interview guide.

Categories	Subcategories
Leadership	Destructive Leadership Constructive leadership
Organizational dimensions	Recognition Role stress (overload, conflict, ambiguity)
Social inequality	Gender inequality
Welfare implications	Discomfort Well-being

## Data Availability

The data are available for peer review upon request and with the authors’ permission.
